# Rhizosphere Bacterial Communities of Two Coastal Halophytes Under Salinity–Flooding Stress

**DOI:** 10.3390/plants15172565

**Published:** 2026-08-24

**Authors:** Zhangchen Xianyu, Shaowei Qin, Dong Li, Dong Wang, Zishuo Wang, Guy Smagghe, Ying Xue, Hualing Xu, Yunpeng Gai

**Affiliations:** 1 School of Grassland Science, Beijing Forestry University, Beijing 100083, China; xy470240647@bjfu.edu.cn (Z.X.); qinshaowei@bjfu.edu.cn (S.Q.); lidong318@bjfu.edu.cn (D.L.); kingzishuo@bjfu.edu.cn (Z.W.); xuexue@bjfu.edu.cn (Y.X.); 2 College of Horticulture and Plant Protection, Inner Mongolia Agricultural University, Hohhot 010018, China; wangdong@imau.edu.cn; 3 Department of Biology, Vrije Universiteit Brussel (VUB), 1050 Brussels, Belgium; guysma9@gmail.com; 4 Institute of Entomology, Guizhou University, Guiyang 550025, China; 5 Dongying Academy of Agricultural Sciences, Dongying 257000, China; yszw2007@sina.com

**Keywords:** halophytes, rhizosphere microbiome, salinity-flooding conditions, bacterial community patterns, consensus association networks, predicted functional potential, coastal wetland restoration

## Abstract

Soil salinisation and periodic flooding jointly shape coastal wetland ecosystems, but how coexisting halophytes differ in their rhizosphere bacterial communities under these conditions remains insufficiently understood. Here, we investigated rhizosphere bacterial communities associated with *Suaeda glauca* Bunge and *Tamarix chinensis* Lour. in saline–alkaline habitats of the Yellow River Delta, China. Twenty quadrat-level rhizosphere samples were collected across four plant–habitat groups, and near-full-length 16S rRNA gene amplicons were sequenced using Pacific Biosciences single-molecule real-time sequencing. Our analysis revealed that hydrological habitat and plant identity together contributed to differences in rhizosphere bacterial community composition. Across the dataset, 2325 bacterial operational taxonomic units were identified. *T. chinensis* showed higher Shannon and Gini–Simpson diversity, whereas richness patterns depended on habitat and the metric examined. Meanwhile, exploratory genus-level association networks revealed host- and habitat-dependent differences in node number, network density and average degree. PICRUSt2-based functional prediction suggested contrasting predicted functional response patterns: the *S. glauca* rhizosphere showed 19 significantly altered predicted pathways between flooded and non-flooded habitats, whereas the *T. chinensis* rhizosphere showed no significant pathway shifts after multiple-testing correction. These findings suggest that coexisting halophytes are associated with divergent rhizosphere bacterial community patterns under saline–alkaline and flooding-associated habitat conditions.

## 1. Introduction

Global soil salinisation is a major environmental challenge affecting terrestrial ecosystems in the 21st century, with impacts extending beyond agricultural productivity to ecosystem stability and biogeochemical cycles [1,2,3]. Recent estimates indicate that salt-affected lands cover more than one billion hectares worldwide, and their extent continues to expand across arid, semi-arid and coastal regions [4,5]. The spread of salt-affected soils contributes to ecological degradation, threatening natural ecosystems and agricultural systems that underpin global food security. Climate change, altered precipitation regimes, warming, sea-level rise, intensive irrigation and coastal development can jointly accelerate salinisation across diverse geographical regions. Without effective mitigation, the extent and severity of soil salinisation are expected to increase [2], with consequences for food production, soil health and ecosystem services [1,6].

Coastal saline–alkaline ecosystems are shaped not only by salinity but also by hydrological processes such as tidal flooding, redox fluctuation and nutrient redistribution. Inundation can alter oxygen availability, ion mobility, soil pH, nutrient pools and organic matter turnover, thereby changing the environmental template in which roots and microorganisms interact [7,8]. For native halophytes, flooding may therefore be interpreted as a hydrological and biogeochemical filter rather than as a uniformly detrimental “stress” in the conventional agronomic sense. This distinction is important because halophytes naturally dominate these habitats, whereas their associated rhizosphere microbiomes may still differ in how they assemble under shared environmental filtering [9,10,11].

China contains extensive salt-affected land, and the Yellow River Delta is a representative coastal saline–alkaline region with strong hydrological gradients and severe soil salinisation [12,13,14,15]. Seawater intrusion and evaporation can increase soil salinity, while tidal flooding and waterlogging can modify oxygen availability and soil nutrient dynamics [13,16,17]. Salt exposure can impair plant photosynthesis, membrane stability, ion homeostasis and growth [18,19]. In parallel, flooding may reduce root-zone oxygen availability and influence root respiration and nutrient acquisition [20,21].

These salinity-inundation dynamics can also influence soil and rhizosphere microbial communities. Previous studies have shown that salinity can alter microbial diversity, abundance, metabolism and nutrient cycling in coastal wetlands and saline soils [22,23]. Rhizosphere microorganisms may affect plant performance through nutrient mobilisation, hormone-related regulation, osmotic adjustment, redox buffering and stress mitigation [24,25,26,27,28,29,30]. Under flooding or hypoxia, some plants may be associated with bacterial taxa that help alleviate oxygen-limitation effects, while under salinity, plant-associated microbes may contribute to ion homeostasis and stress tolerance [31,32].

Plant growth-promoting rhizobacteria (PGPRs) further illustrate the applied ecological relevance of rhizosphere microbial ecology. PGPR inoculation has been shown to mitigate saline–alkaline or flooding constraints in some systems, promoting plant growth under adverse soil conditions [33,34,35,36,37]. Experimental studies suggest that PGPRs can regulate plant osmotic balance and ion homeostasis through hormone modulation, gene expression, protein function and metabolite synthesis [38]. They may also enhance antioxidant activity, osmolyte accumulation, salt compartmentalisation, proton transport and nutrient status, thereby helping to alleviate osmotic stress and ion toxicity [39]. Together, these studies support the broader view that rhizosphere microorganisms are important components of plant adaptation in saline environments and provide an ecological rationale for microbiome-informed restoration.

Halophytes such as *Suaeda glauca* Bunge and *Tamarix chinensis* Lour. are key species in the Yellow River Delta and provide useful systems for examining plant–microbe interactions under saline–alkaline conditions [40,41]. *S. glauca* tolerates high salinity through mechanisms including Na^+^/H^+^ exchange, vacuolar ion sequestration, and selective root uptake [42,43]. Its root exudates promote the enrichment of beneficial rhizobacteria, such as *Gemmata* and *Gemmatimonas*, which contribute to nitrogen fixation, nutrient availability, and soil structure improvement, thereby enhancing plant salt tolerance [44,45]. Similarly, *T. chinensis* exhibits strong salt tolerance and can influence rhizosphere soil properties and microbial recruitment in saline environments [46,47,48]. Importantly, comparing these two species is not an attempt to treat them as ecologically identical hosts; rather, it provides an explicit host-identity comparison under the same regional salinity–flooding filter. Their contrasting life-history strategies, growth forms, and salt-handling traits make them an ecologically informative pair for examining whether host identity modifies rhizosphere microbiome assembly under shared external conditions. Despite these insights, knowledge gaps remain regarding whether coexisting halophytes maintain similar rhizosphere bacterial communities under flooded and non-flooded saline–alkaline conditions or instead harbour distinct microbiome assembly patterns. High-throughput amplicon sequencing and related bioinformatic approaches now allow detailed characterisation of these communities, providing opportunities to examine microbial community patterns and predicted functional potential in environmental adaptation and ecosystem restoration [49,50].

In this study, we collected rhizosphere soil samples from *S. glauca* and *T. chinensis* in flooded and non-flooded saline–alkaline habitats of the Yellow River Delta. We used PacBio single-molecule real-time near-full-length 16S rRNA gene amplicon sequencing to compare bacterial community composition, alpha and beta diversity, taxonomic signatures, predicted functional profiles, exploratory genus-level association networks, biomarker taxa and descriptive phylogenetic patterns. We hypothesised that both hydrological habitat and plant identity would contribute to differences in bacterial community composition. Because the flooded and non-flooded conditions were represented by different field sites, and each treatment group comprised only five biological replicates, we interpret the results as habitat-associated patterns and avoid strong causal attribution to flooding alone. Consequently, the genus-level consensus association networks are regarded as exploratory and hypothesis-generating rather than definitive ecological interaction networks. The findings provide preliminary insights into halophyte-associated microbiomes and their potential relevance for future microbiome-informed saline–alkaline wetland restoration.

## 2. Results

### 2.1. Descriptive Differences in Soil Physicochemical Properties Between Flooded and Non-Flooded Rhizosphere Habitats

**Figure 1:**
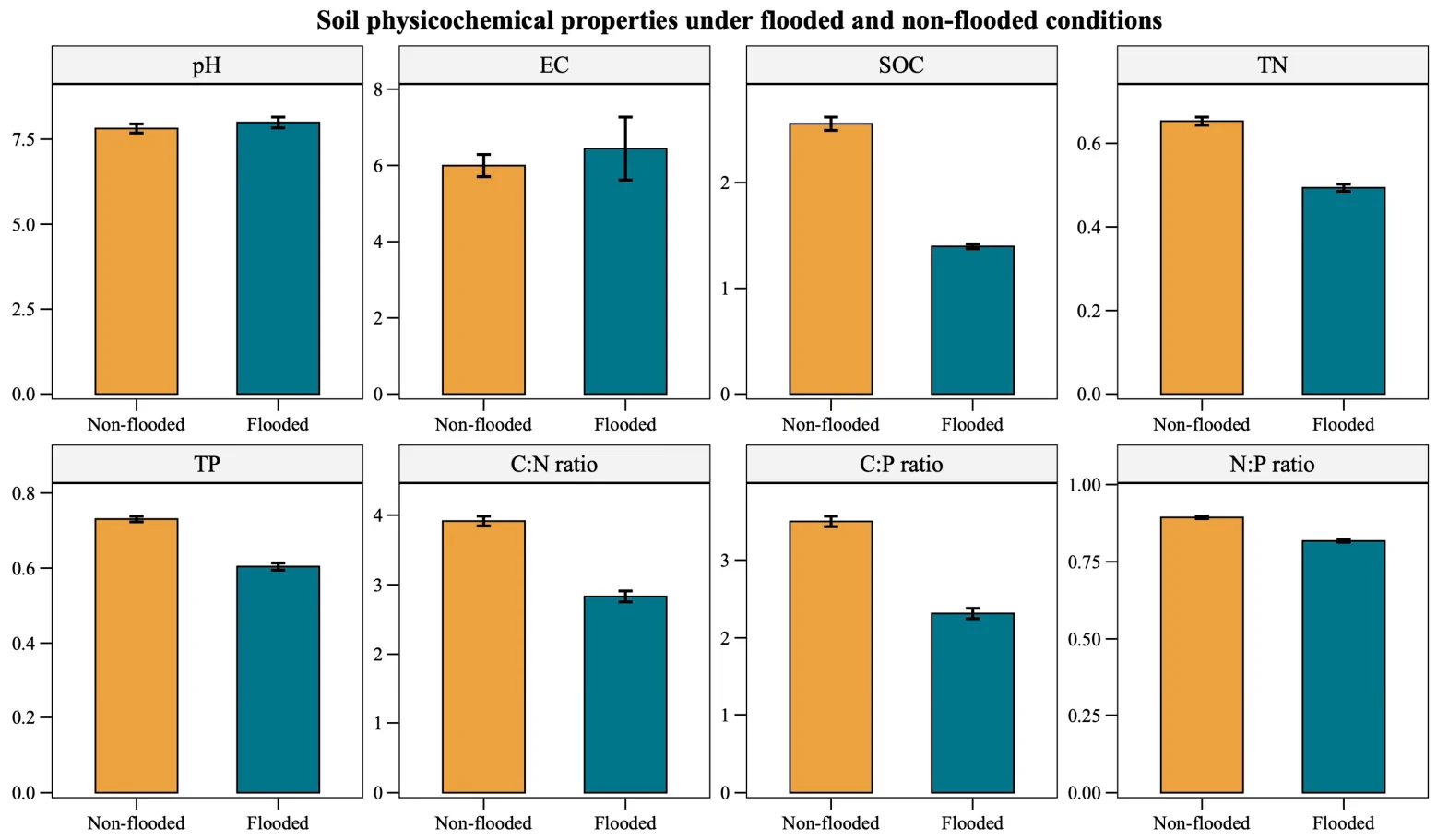
Descriptive comparison of soil physicochemical properties under flooded and non-flooded conditions. Bars represent quadrat-level group means, and error bars represent standard deviations.

Soil physicochemical properties showed clear descriptive differences between the flooded and non-flooded saline–alkaline rhizosphere habitats (Figure 1). The flooded habitat showed slightly higher pH and EC than the non-flooded habitat. Mean pH was 7.990 ± 0.158 in the flooded habitat and 7.814 ± 0.133 in the non-flooded habitat. Mean EC was 6.442 ± 0.826 mS cm^−1^ in the flooded habitat and 5.996 ± 0.291 mS cm^−1^ in the non-flooded habitat. These descriptive patterns indicate that the flooded rhizosphere habitat was characterised by slightly stronger alkalinity and higher soluble salt levels. In contrast, nutrient-related variables were consistently higher in the non-flooded habitat. SOC was 2.557 ± 0.063 g kg^−1^ in the non-flooded habitat and 1.397 ± 0.023 g kg^−1^ in the flooded habitat. TN was 0.653 ± 0.010 g kg^−1^ under non-flooded conditions and 0.494 ± 0.009 g kg^−1^ under flooded conditions. TP was also higher in the non-flooded habitat, with values of 0.731 ± 0.008 g kg^−1^ compared with 0.604 ± 0.010 g kg^−1^ in the flooded habitat.

Stoichiometric ratios displayed a similar descriptive pattern. The non-flooded habitat had higher C:N, C:P, and N:P ratios, with values of 3.915 ± 0.070, 3.499 ± 0.067, and 0.894 ± 0.004, respectively. The corresponding values in the flooded habitat were 2.831 ± 0.079, 2.314 ± 0.067, and 0.817 ± 0.004. These descriptive differences suggest that the sampled non-flooded habitat had higher SOC, TN, and TP contents, as well as higher C:N, C:P, and N:P ratios, than the sampled flooded habitat. Overall, the soil physicochemical data indicate a distinct habitat-associated contrast between flooded and non-flooded saline–alkaline rhizospheres. Detailed soil physicochemical summary statistics are provided in Table S1.

### 2.2. Plant–Habitat-Associated Variation in Rhizosphere Bacterial Alpha Diversity

**Figure 2:**
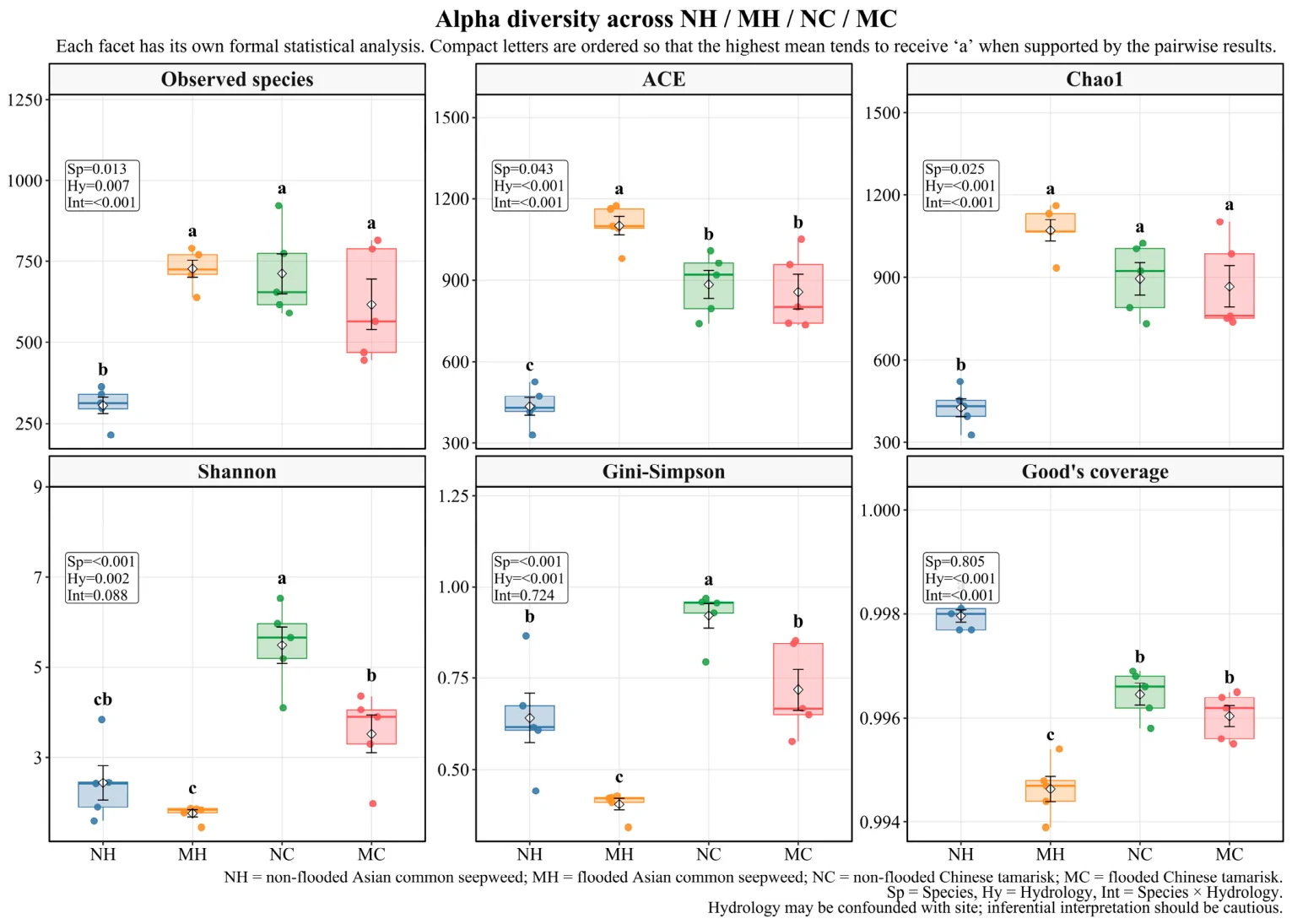
Alpha diversity of rhizosphere bacterial communities across NH, MH, NC, and MC. Boxplots show observed species richness, ACE, Chao1, Shannon diversity, Gini–Simpson diversity, and Good’s coverage for NH, non-flooded *Suaeda glauca*; MH, flooded *S. glauca*; NC, non-flooded *Tamarix chinensis*; and MC, flooded *T. chinensis*. Different lowercase letters indicate groups that differed significantly in the pairwise comparisons shown in the figure (*p* < 0.05); groups sharing a letter were not significantly different.

Alpha-diversity analysis showed clear plant–habitat-associated variation in rhizosphere bacterial richness, diversity, and sequencing coverage across NH, MH, NC, and MC (Figure 2). The evaluated metrics included observed species richness, ACE, Chao1, Shannon diversity, Gini–Simpson diversity, and Good’s coverage. Flooded habitats were associated with reduced bacterial diversity across both plant species, but the magnitude of reduction was species-dependent. Under non-flooded conditions, the rhizosphere of *T. chinensis* supported the highest bacterial diversity (Shannon index: 5.49 ± 0.91; Gini–Simpson index: 0.92 ± 0.07), significantly exceeding that of *S. glauca*. In contrast, *S. glauca* displayed the lowest bacterial richness under non-flooded soils (Chao1: 424 ± 71; ACE: 433 ± 72), suggesting a limited bacterial recruitment potential. Under flooding stress, community responses differed markedly between species. While *T. chinensis* experienced substantial diversity reductions (~35% decrease in Shannon index), it maintained significantly higher diversity than *S. glauca*. Interestingly, *S. glauca* exhibited increased richness under flooding (Chao1: 1072 ± 87; ACE: 1102 ± 77), possibly reflecting reduced competitive exclusion or altered niche availability in response to stress. Cross-species comparisons showed that *T. chinensis* had higher Shannon and Gini–Simpson diversity than *S. glauca* under the sampled conditions. Across both environmental conditions, *T. chinensis* showed higher bacterial diversity than *S. glauca*, whereas differences in estimated richness depended on the hydrological habitat and the diversity metric examined.

### 2.3. Hydrological Habitat and Plant Identity Jointly Shape Rhizosphere Bacterial Community Composition

**Figure 3:**
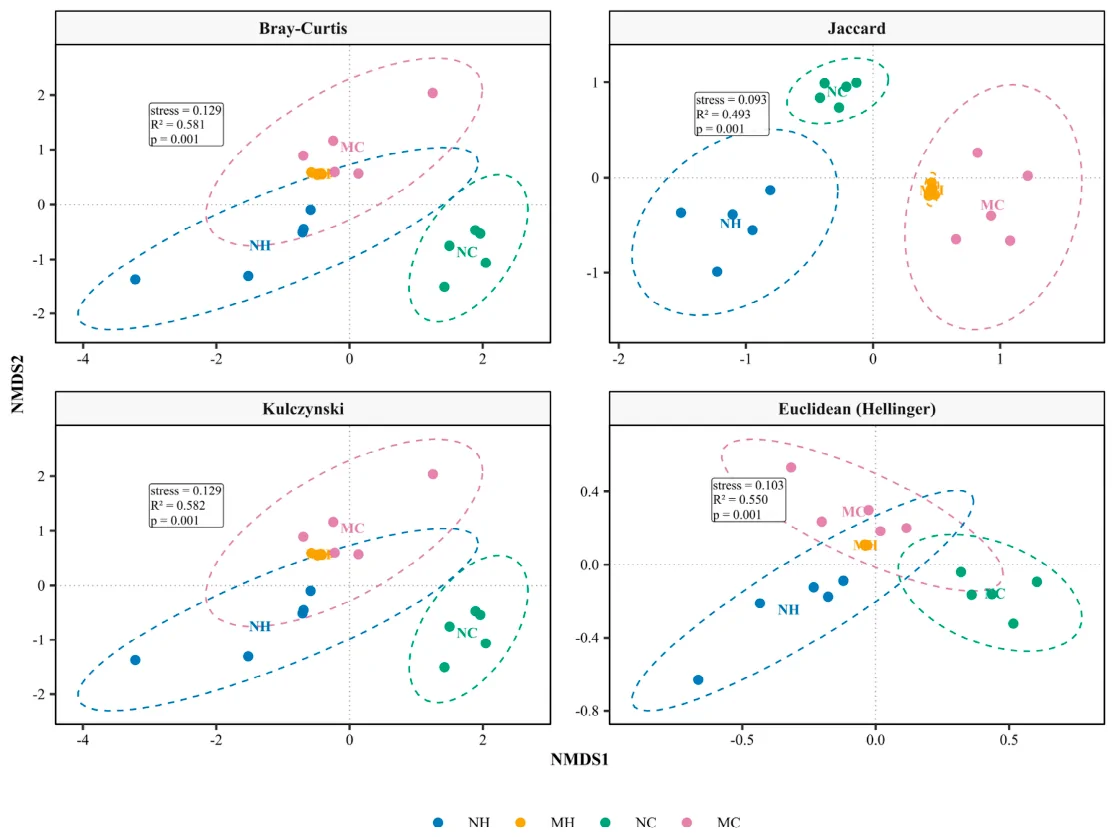
Sensitivity analysis of NMDS patterns across four dissimilarity strategies. Panels show NMDS ordinations of rhizosphere bacterial communities based on Bray–Curtis, binary Jaccard, Kulczynski, and Euclidean distance after Hellinger transformation. Points represent individual samples, dashed ellipses indicate 95% group envelopes, and group labels are placed at centroids.

Beta diversity analysis using non-metric multidimensional scaling (NMDS) based on four dissimilarity metrics (Bray–Curtis, Jaccard, Kulczynski, and Euclidean distance after Hellinger transformation) revealed distinct patterns of bacterial community assembly across conditions (Figure 3). PERMANOVA confirmed statistically significant differences in community composition among all condition groups, with both flooding status and plant species identity contributing significantly to variation. Notably, the non-flooded *T. chinensis* group (NC) occupied the most distinct position in ordination space, exhibiting the greatest compositional divergence from all other conditions. This suggests that in the non-flooded habitat, *T. chinensis* recruits and maintains a highly specialised bacterial assemblage that differs substantially from communities associated with flooding stress and alternative plant hosts. 

### 2.4. Taxonomic Composition and OTU Overlap Reveal Host- and Habitat-Associated Rhizosphere Bacterial Patterns

**Figure 4:**
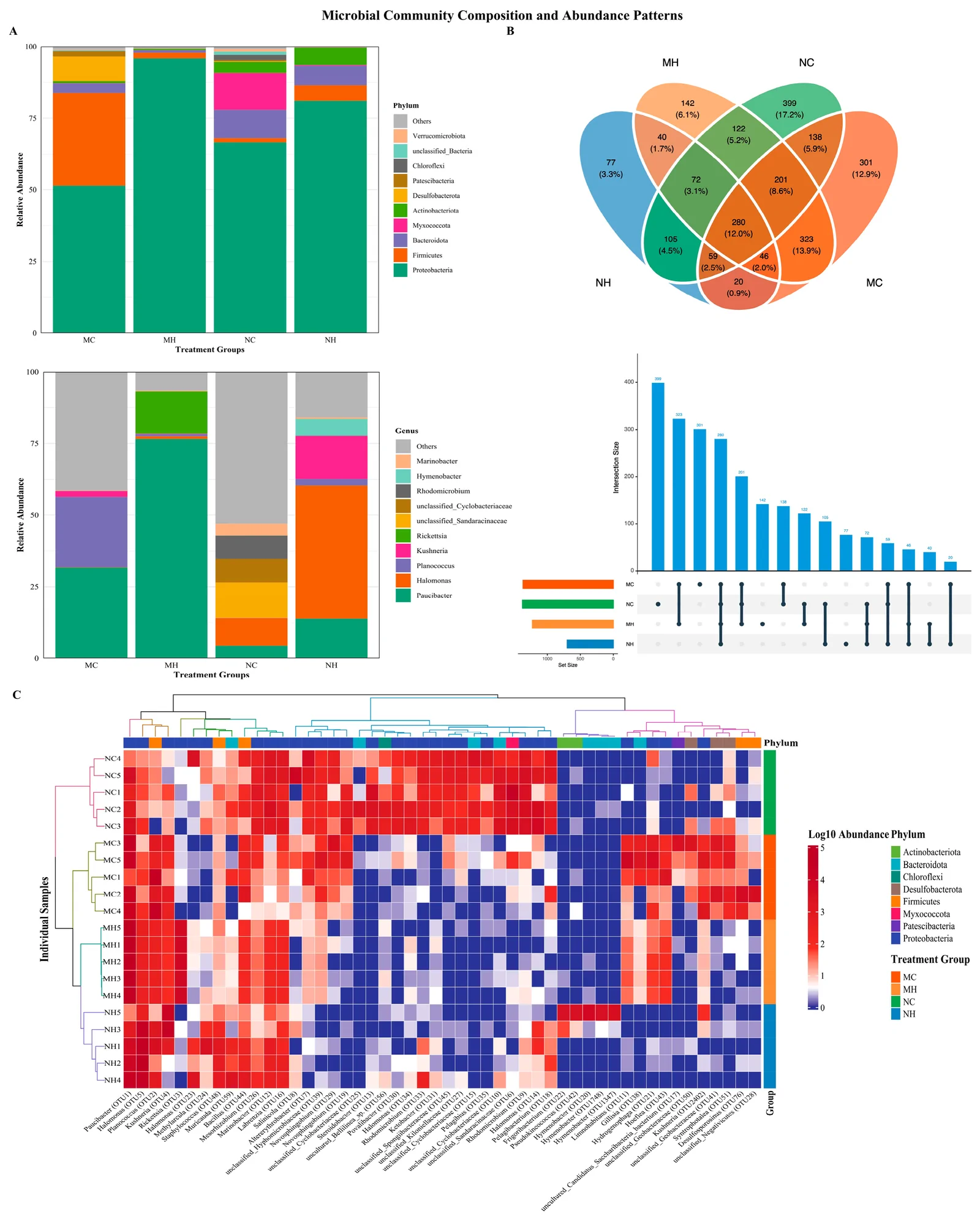
Taxonomic composition and descriptive OTU overlap patterns of rhizosphere bacterial communities associated with *S. glauca* and *T. chinensis* under flooded and non-flooded saline–alkaline conditions. (**A**) Stacked bar plots showing the relative abundances of the dominant bacterial phyla (top) and genera (bottom) across the four condition groups. (**B**) Descriptive set-intersection analysis of OTU membership among NH, MH, NC, and MC after within-group prevalence filtering; the upper panel shows the four-set overlap diagram, and the lower panel shows the corresponding UpSet summary of intersection size and set size. In the analysis workflow, OTU presence within a condition was defined as detection in at least two biological replicates with a per-sample count ≥ 1. This panel is descriptive and should not be interpreted as formal differential-abundance inference. (**C**) Heatmap of the 50 most abundant genera based on log10-transformed relative abundance, with hierarchical clustering applied to both samples and genera. The side annotation indicates condition group, and the upper annotation indicates phylum affiliation of the displayed genera. NH, non-flooded *S. glauca*; MH, flooded *S. glauca*; NC, non-flooded *T. chinensis*; MC, flooded *T. chinensis*.

Taxonomic profiling showed clear host- and habitat-associated differences in rhizosphere bacterial community composition across the four plant–habitat groups (Figure 4A). Proteobacteria was the dominant phylum in all groups, with its relative abundance ranging from 51.38% to 95.50%. Under flooding, the *S. glauca* rhizosphere exhibited extreme Proteobacteria dominance (95.50%), which is a 14.43% increase compared to non-flooded conditions (81.07%). In contrast, *T. chinensis* showed a decrease in Proteobacteria from 66.49% (non-flooded) to 51.38% (flooded), highlighting species-specific responses to anaerobic stress.

Firmicutes displayed an inverse relationship with flooding across plant species. In *T. chinensis*, the relative abundance of Firmicutes was higher in the flooded group (32.44%) than in the non-flooded group (1.53%). In *S. glauca,* Firmicutes accounted for 5.41% and 1.94% of the bacterial communities in the non-flooded and flooded groups, respectively. Actinobacteriota and Bacteroidota accounted for 3.83–6.06% and 6.84–9.88%, respectively, in the non-flooded groups, whereas their relative abundances were lower in the flooded groups.

At the genus level, 187–326 genera were detected per group, and the relative-abundance profiles of dominant taxa differed descriptively among the four plant–habitat groups. *S. glauca* under flooding exhibited a simplified structure dominated by *Paucibacter* (76.51%) and *Rickettsia* (14.74%), together accounting for >90% of total abundance. This abundance pattern indicates that the flooded *S. glauca* group was dominated by a small number of genera. The flooded *T. chinensis* rhizosphere communities were more taxonomically diverse, harbouring 14 dominant genera, including *Paucibacter* (31.73%), *Planococcus* (24.45%), *Desulfosporosinus* (3.24%), and *Limnohabitans* (3.20%).

Under non-flooded conditions, both species supported more equitable genus distributions. *S. glauca* was dominated by halotolerant genera, including *Halomonas* (46.45%), *Kushneria* (14.90%), and *Paucibacter* (13.91%), reflecting adaptation to saline soils. *T. chinensis* displayed the highest taxonomic evenness, with 21 dominant genera such as *Sandaracinaceae* (12.31%), *Halomonas* (9.96%), *Cyclobacteriaceae* (8.24%), and *Rhodomicrobium* (8.13%). Comparative abundance analysis revealed that *Paucibacter* increased by 62.60 and 27.29 percentage points in the *S. glauca* and *T. chinensis* comparisons, respectively, suggesting key adaptive traits for oxygen-limited environments, whereas aerobic-associated taxa such as *Halomonas* decreased substantially (a decrease of 45.16 percentage points in *S. glauca*), reflecting sensitivity to redox changes.

Community-level diversity assessed through OTU analysis identified 2,325 OTUs across the dataset (Figure 4B). At the group level, more OTUs were detected in the *T. chinensis* groups than in the corresponding *S. glauca* groups under both flooded and non-flooded conditions; however, these cumulative group-level OTU counts should not be interpreted as sample-level richness. Set-overlap analysis showed that 12.0% of the prevalence-filtered OTUs were shared across all four conditions, indicating minimal core microbiome overlap and strong ecological filtering by plant–environment interactions.

Hierarchical clustering of the 50 most abundant genera revealed three distinct assemblages corresponding to plant species and flooding regime (Figure 4C). *S. glauca* samples clustered together regardless of flooding, indicating strong host-plant control over community assembly. Within-species clustering was further modulated by flooding, forming subclusters characterised by specific biomarker genera, including *Marinobacter*, *Labrenzia*, and *Rickettsia* in *S. glauca* and *Altererythrobacter*, *Novosphingobium*, and *Hydrogenophaga* in *T. chinensis*. These results demonstrate that both flooding and plant identity significantly shape rhizosphere bacterial community structure, with *T. chinensis* consistently supporting greater diversity and taxonomic richness than *S. glauca* across both environmental conditions.

### 2.5. Predicted Functional Profiles Differ Between Hydrological Habitats in S. glauca but Not Significantly in T. chinensis

**Figure 5:**
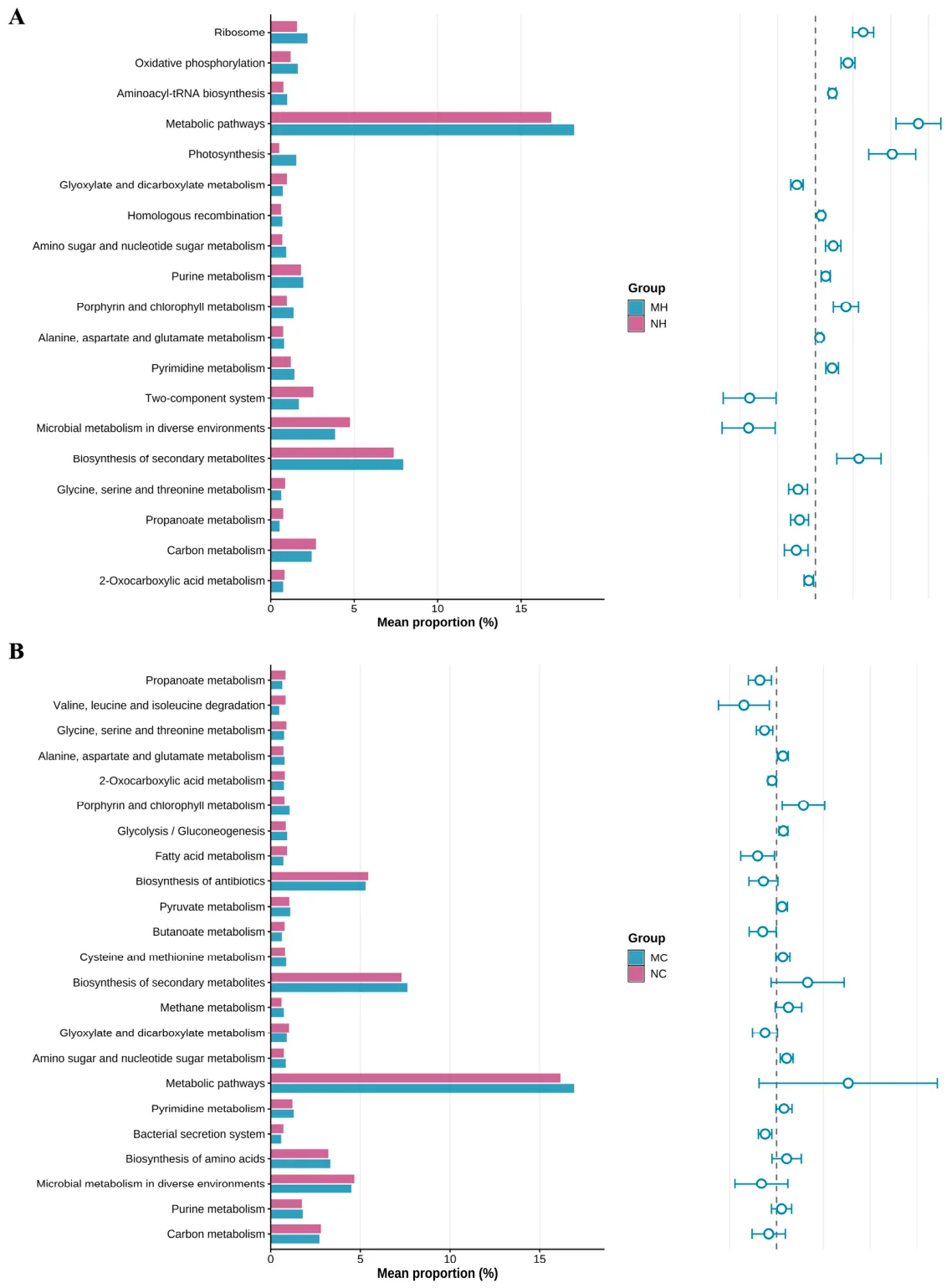
Host-dependent responses of predicted rhizosphere functional potential to flooding. PICRUSt2-based KEGG level 3 pathway profiles were compared between flooded and non-flooded rhizosphere bacterial communities associated with (**A**) *S. glauca* (MH vs. NH) and (**B**) *T. chinensis* (MC vs. NC). In each panel, the left subpanel shows the mean relative abundance (%) of each pathway in the two groups, the middle subpanel shows the difference between group means with 95% confidence intervals, and the right subpanel shows the multiple-testing-adjusted *p* values. The vertical dashed line indicates no difference between groups.

PICRUSt2-based analysis indicated contrasting patterns in predicted functional potential between hydrological habitats for the two halophytes; these predictions do not directly measure metabolic activity. The predicted functional profiles of the *S. glauca* rhizosphere differed between the non-flooded (NH) and flooded (MH) groups, with 19 predicted pathways showing significant differences in relative abundance after multiple-testing correction (Figure 5A). Several predicted pathways related to protein synthesis and energy metabolism had higher relative abundances in MH than in NH. For example, ribosome biogenesis, oxidative phosphorylation, and aminoacyl-tRNA biosynthesis all showed higher predicted relative abundance (*p* = 0.002). Additionally, predicted pathways related to secondary metabolite biosynthesis and photosynthesis showed higher relative abundances in MH than in NH (*p* = 0.005). Conversely, glyoxylate and dicarboxylate metabolism showed a lower predicted relative abundance in MH than in NH (*p* = 0.005).

In contrast, *T. chinensis* showed no statistically significant differences in the analysed predicted pathways between non-flooded (NC) and flooded (MC) conditions (Figure 5B). Among 23 analysed pathways, none reached statistical significance (all adjusted *p* ≥ 0.09). Pathways such as methane metabolism and secondary metabolite biosynthesis also remained non-significant (*p* = 0.14 each); thus, no pathway-level difference was detected in this comparison.

Taken together, the two host species showed contrasting habitat-associated patterns in predicted functional potential: 19 pathways differed between NH and MH for *S. glauca*, whereas no pathway reached significance after multiple-testing correction between NC and MC for *T. chinensis*.

These results support a difference in predicted functional response patterns between the two host-associated rhizosphere communities. Because PICRUSt2 infers functional potential from marker-gene data, the pathway differences should be interpreted as predictions and require validation with direct functional measurements.

### 2.6. Host- and Habitat-Associated Differences in Microbial Association Networks

**Figure 6:**
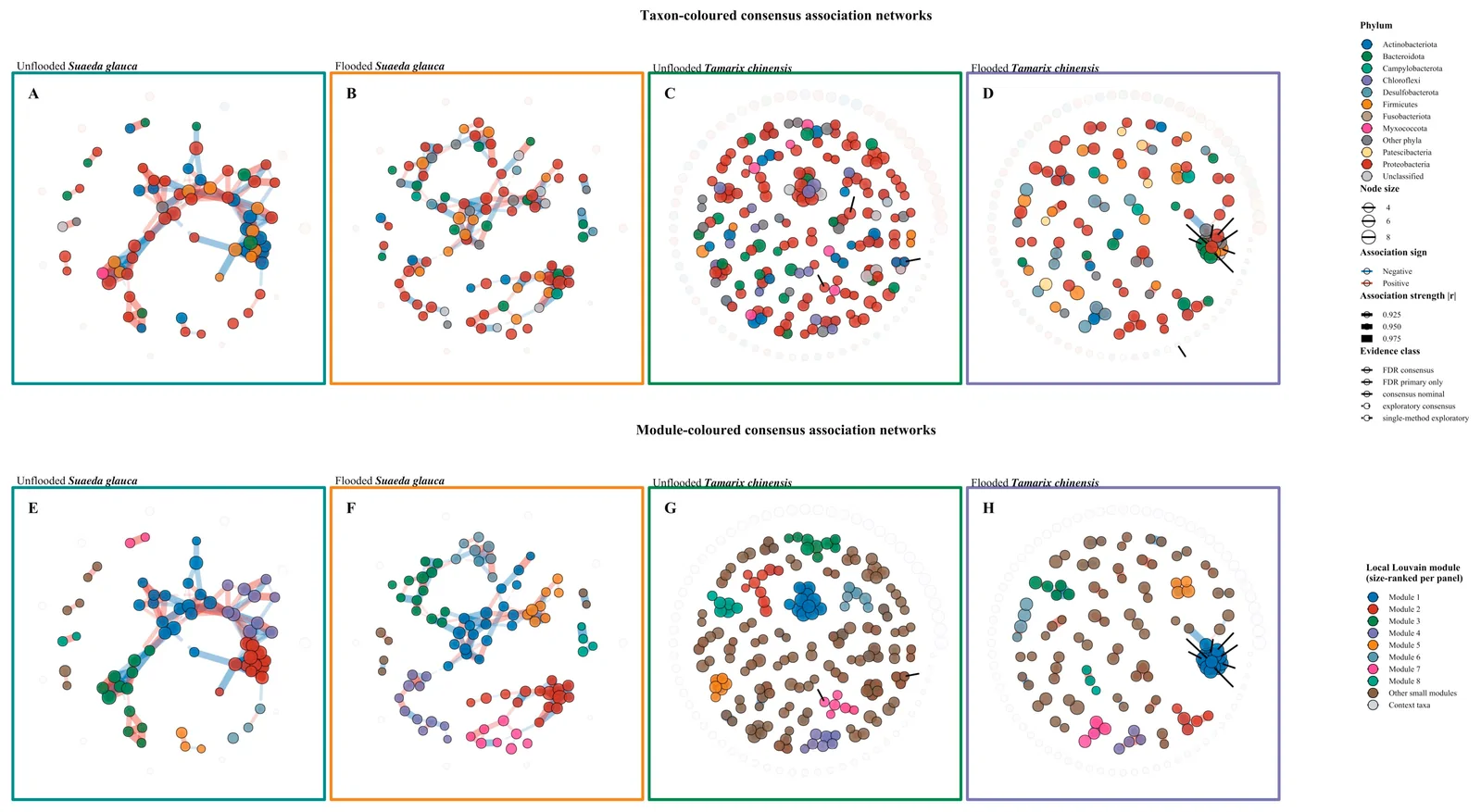
Treatment-specific microbial consensus association networks inferred at the genus level for rhizosphere bacterial communities under contrasting flooding regimes. Panels (**A**–**D**) show phylum-coloured consensus display networks for NH, non-flooded *Suaeda glauca*; MH, flooded *S. glauca*; NC, non-flooded *Tamarix chinensis*; and MC, flooded *T. chinensis*, respectively. Panels (**E**–**H**) show the corresponding Louvain module-coloured networks using the same layouts. Edge evidence classes are indicated by line style: solid lines denote FDR-supported associations (Benjamini–Hochberg FDR < 0.05) passing the consensus filter (Pearson |r| ≥ 0.65, Spearman sign concordance, leave-one-out sign consistency ≥ 0.60); dashed lines denote display-layer edges meeting the |r| ≥ 0.65 and sign-consistency thresholds but not surviving FDR correction; and dotted lines denote context-only taxon pairs below the display threshold. Faint grey halo nodes represent context-only taxa with degree zero in the inferred network.

Genus-level consensus association network analysis showed host- and habitat-associated differences in exploratory rhizosphere bacterial association structures between *S. glauca* and *T. chinensis* (Figure 6). The networks were constructed from CLR-transformed genus-level abundance profiles using primary Pearson correlation, secondary Spearman sign screening, leave-one-out sign-consistency assessment and Benjamini–Hochberg FDR correction.

In the balanced consensus display layer, the non-flooded *S. glauca* network contained 78 displayed nodes and 180 displayed edges, including 18 FDR-supported edges, with a density of 0.060 and an average degree of 4.62. The flooded *S. glauca* network contained 103 displayed nodes and 180 displayed edges, including one FDR-supported edge, with a density of 0.034 and an average degree of 3.50. Thus, the flooded *S. glauca* habitat showed a larger displayed taxon set but lower density, lower average degree and fewer FDR-supported associations. The non-flooded *T. chinensis* network contained 237 displayed nodes and 180 displayed edges, including 10 FDR-supported edges, with a density of 0.006 and an average degree of 1.52. The flooded *T. chinensis* network contained 179 displayed nodes and 180 displayed edges, including 61 FDR-supported edges, with a density of 0.011 and an average degree of 2.01. Thus, the flooded *T. chinensis* habitat showed fewer displayed taxa but higher density, higher average degree and more FDR-supported associations.

### 2.7. Random Forest and LEfSe Identify Distinct Plant–Habitat-Associated Biomarker Signatures

**Figure 7:**
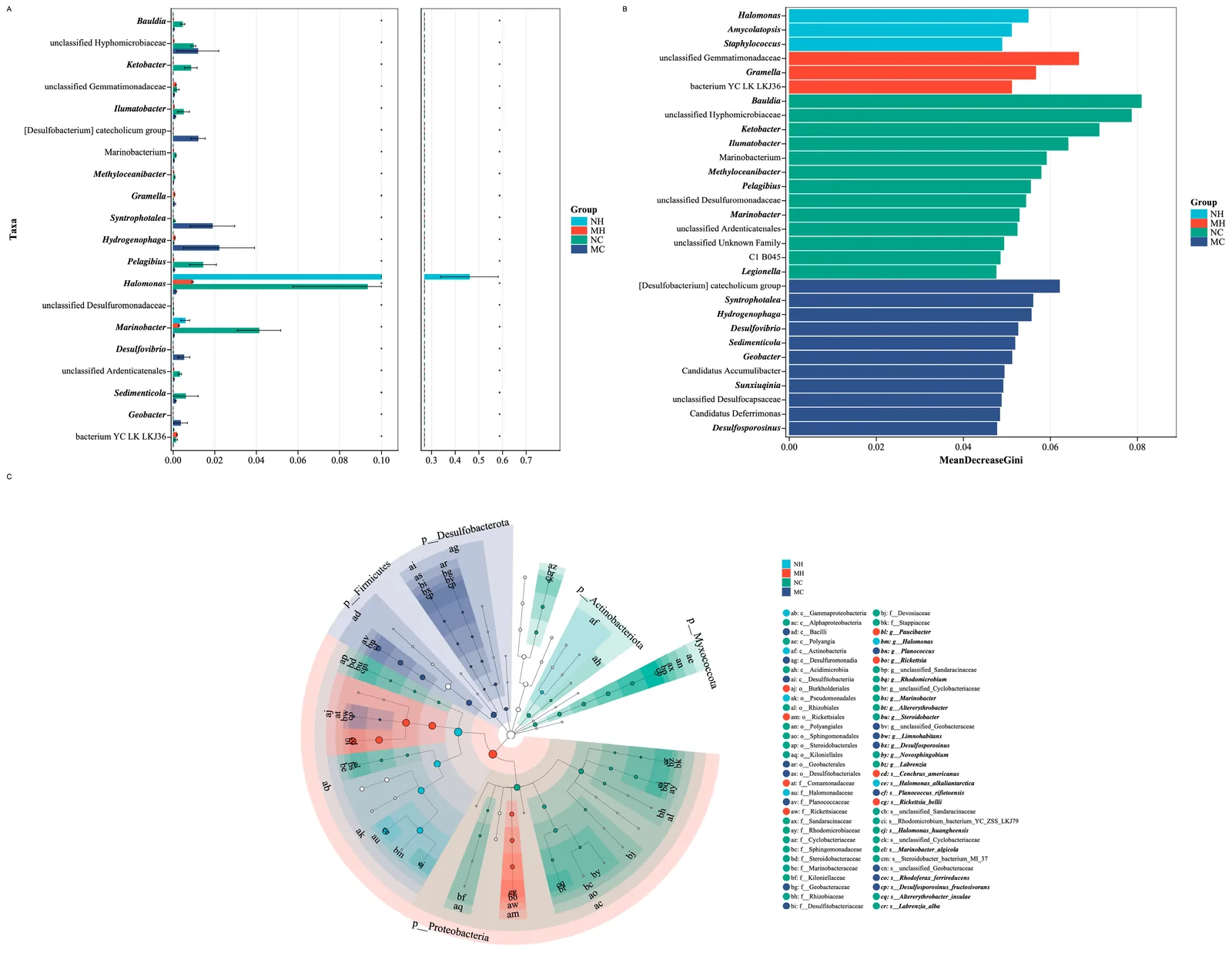
Random forest and LEfSe identification of plant–habitat-associated rhizosphere bacterial signatures. (**A**) Group-associated relative-abundance patterns of the top 20 genus-level discriminative taxa from the RF/non-parametric workflow. Asterisks indicate significant group differences after Benjamini–Hochberg correction (adjusted *p* < 0.05). A two-segment abundance axis is used only to display low- and high-abundance ranges and does not alter the underlying abundance or statistical results. (**B**) Feature-importance ranking of the top 30 genus-level taxa by MeanDecreaseGini; colours denote the associated plant–habitat group. (**C**) All-rank LEfSe taxonomic cladogram restricted to features with LDA > 4.0. Coloured nodes and sectors indicate group-associated lineages, while higher taxonomic ranks are labelled directly and lower ranks are linked to the letter-coded two-column legend. NH, non-flooded *S. glauca*; MH, flooded *S. glauca*; NC, non-flooded *T. chinensis*; MC, flooded *T. chinensis*.

To identify genus-level taxa that best discriminated among the four plant–habitat groups, we applied a random forest (RF) workflow coupled with non-parametric significance testing. The top 30 discriminative taxa ranked by MeanDecreaseGini are shown in Figure 7B, and all 30 displayed taxa had Benjamini–Hochberg (BH)-adjusted *p* values < 0.05 in the corresponding differential-analysis results. The three highest-ranked features were *Bauldia* (NC; MeanDecreaseGini = 0.0809), unclassified Hyphomicrobiaceae (NC; 0.0786), and *Ketobacter* (NC; 0.0712), followed by unclassified Gemmatimonadaceae (MH; 0.0665) and *Ilumatobacter* (NC; 0.0641). Among the top 30 RF features, 13 were associated with NC, 11 with MC, and three each with NH and MH, indicating that most high-ranking discriminative genera were assigned to the two *T. chinensis*-associated groups. Figure 7A shows the group-associated relative-abundance patterns of the top 20 RF features. These included *Halomonas* in NH; unclassified Gemmatimonadaceae, *Gramella*, and bacterium YC LK LKJ36 in MH; *Bauldia*, unclassified Hyphomicrobiaceae, *Ketobacter*, *Ilumatobacter*, *Marinobacterium*, *Methyloceanibacter*, *Pelagibius*, unclassified Desulfuromonadaceae, *Marinobacter*, and unclassified Ardenticatenales in NC; and [Desulfobacterium] catecholicum group, *Syntrophotalea*, *Hydrogenophaga*, *Desulfovibrio*, *Sedimenticola*, and *Geobacter* in MC.

An independent all-rank LEfSe analysis provided a complementary taxonomic view of the condition-associated signatures (Figure 7C). After BH correction, 70 taxonomic features with LDA scores strictly greater than 4.0 were retained for the cladogram; these features were assigned to NC (37), MC (18), MH (9), and NH (6). The NH signature included Gammaproteobacteria, Pseudomonadales, Halomonadaceae, and *Halomonas*, whereas the MH signature included Proteobacteria, Burkholderiales, Comamonadaceae, and *Paucibacter*. The MC signature included Firmicutes, Bacilli, Planococcaceae/*Planococcus*, and *Geobacter*- and *Desulfosporosinus*-associated lineages. In contrast, NC contained a broad Alphaproteobacteria/Rhizobiales-associated signature together with lineages containing *Marinobacter*, *Altererythrobacter*, *Steroidobacter*, *Rhodomicrobium*, *Novosphingobium*, and *Labrenzia*. Because parent and child taxonomic ranks are represented simultaneously in the LEfSe cladogram, these feature counts describe nested taxonomic signatures rather than independent biological entities. Together, the genus-level RF results and the multi-rank LEfSe analysis consistently identified distinct taxonomic signatures associated with the four sampled plant–habitat groups.

### 2.8. Descriptive Phylogenetic Patterns in Bacterial Community Structure

**Figure 8:**
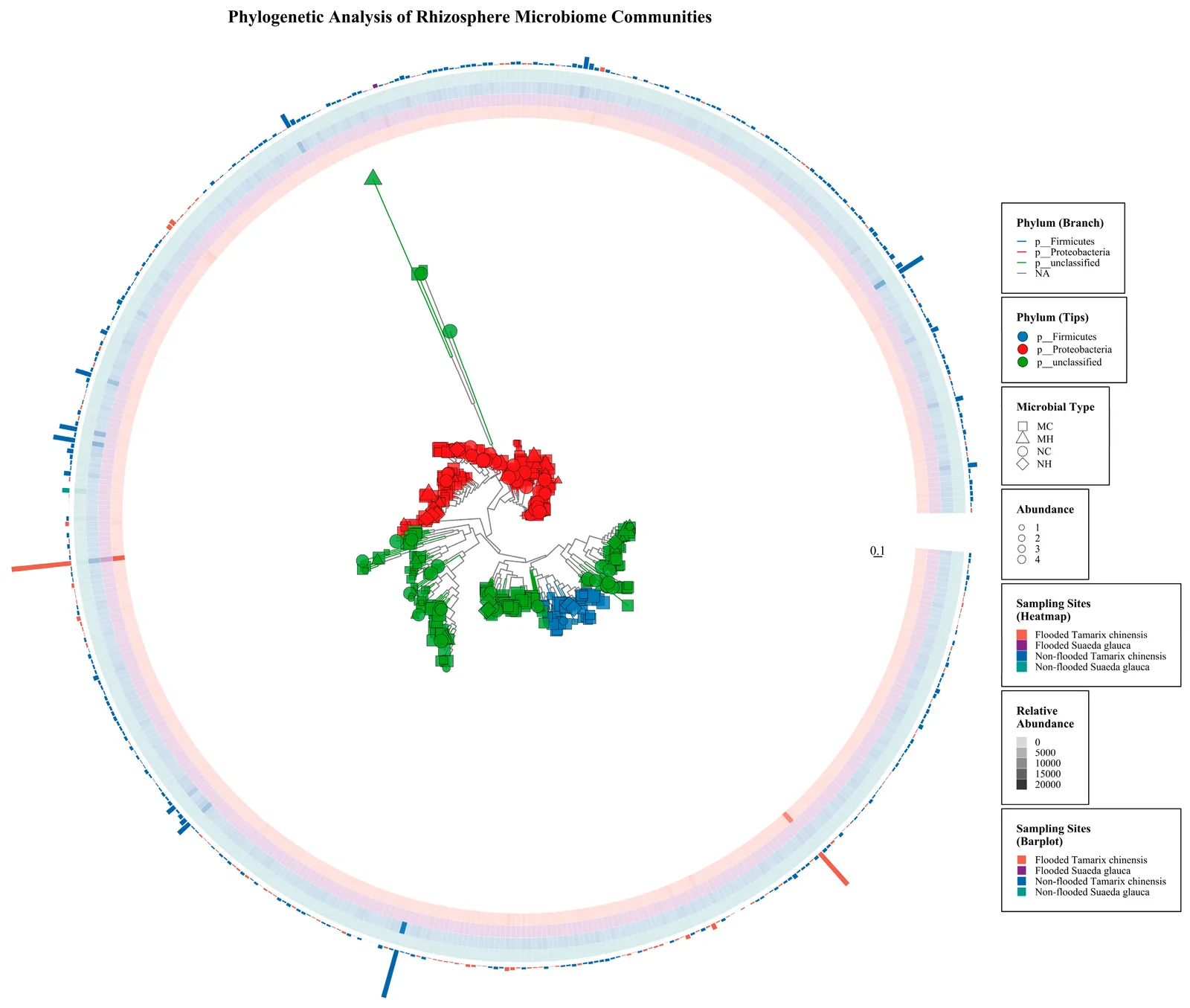
Phylogenetic analysis of rhizosphere bacterial communities across different plant species and flooding conditions. The circular phylogenetic tree displays the evolutionary relationships among the top 500 OTUs identified across all the sampling sites.

Phylogenetic analysis of the top 500 OTUs across all sampling sites revealed distinct evolutionary patterns and taxonomic clustering within the rhizosphere bacterial communities (Figure 8). The circular phylogenetic tree highlighted clear segregation of bacterial taxa into major lineages, with Proteobacteria dominating the tree, followed by Firmicutes and several unclassified groups.

The circular tree showed host- and habitat-associated distribution patterns among the 500 most abundant OTUs. Non-flooded *T. chinensis* samples (NC) were represented across multiple major clades, whereas MC-associated abundances were visually concentrated in a subset of lineages. The accompanying heatmap and outer bar plot displayed variation in taxon abundance across the four plant–habitat groups. Thus, Figure 8 provides a descriptive phylogenetic context for the taxonomic abundance patterns observed across the sampled plant–habitat groups.

## 3. Discussion

The present study showed that two coexisting halophytes in the Yellow River Delta were associated with distinct rhizosphere bacterial community patterns under saline–alkaline and hydrological habitat contrasts. Hydrological habitat conditions and plant identity together contributed to differences in rhizosphere microbial community composition. These findings represent field-based evidence for understanding plant–microbe associations in saline–alkaline wetland environments and may inform future studies on differential stress tolerance among coexisting halophytic species. Salinisation and flooding are important environmental factors affecting plant growth and development [51]. In saline–alkaline soils, excessive salt concentrations can induce osmotic dehydration in plant cells and lead to ion toxicity that disrupts normal physiological metabolism [52]. Under flooding conditions, root hypoxia can impair root function and create imbalances between photosynthesis and respiration that constrain plant growth [53]. To cope with these stresses, different plant species may be associated with distinct patterns of enrichment or depletion of rhizosphere microbial taxa, thereby shaping community structures that may contribute to stress resistance and plant growth. Our results showed differences in rhizosphere microbiome composition between the two dominant plant species in the Yellow River Delta saline–alkaline wetlands, namely *S. glauca* and *T. chinensis*, under flooded and non-flooded conditions. These results suggest that, within saline–alkali environments, flooded habitats are associated with divergent rhizosphere bacterial patterns in these plants; these patterns may reflect taxon-specific filtering and may contribute to niche-associated microbial assemblages under combined salinity–flooding conditions.

Flooding in coastal saline–alkaline habitats can influence microbial community structure by modifying physicochemical properties in rhizosphere soil, a process of ecological relevance under global environmental change [54]. Previous studies have shown that certain *Limnohabitans* strains are correlated with total phosphorus concentrations in aquatic systems, which provides relevant ecological context for our observations [55]. Additionally, this bacterial group has been reported to respond to dissolved organic matter (DOM) inputs and to possess organic matter metabolic capabilities, which may be related to the lower SOC observed in the sampled flooded habitat [56].

Furthermore, *Kushneria*, a typical halophilic genus, is adapted to high-salinity and oligotrophic environments. Some representative strains within this genus can solubilise organic phosphorus and insoluble inorganic phosphorus, converting them into bioavailable phosphorus [57,58]. *Steroidobacter* represents a group of organic matter-degrading bacteria distributed in soils and may play roles in carbon cycling, particularly in organically enriched habitats where its abundance can be associated with SOC and TN contents. Moreover, *Marinobacter* includes common aerobic heterotrophic bacteria in saline–alkaline soils and marine environments, and some members are classified as hydrocarbon-degrading bacteria [59]. These taxa may contribute to nutrient and elemental cycling through hydrocarbon degradation and related metabolic capabilities [60,61]. The reported functional characteristics of these microorganisms indicate their potential roles in nutrient cycling in coastal saline–alkali land, suggesting that saline–alkali flooding conditions may affect the predicted functional potential of rhizosphere microbial communities by altering soil physicochemical properties.

Under environmental selection pressure, halophytic species may be associated with different rhizosphere microbial assembly patterns that contribute to their adaptation to saline and flooded environments [62]. Our analysis of multiple alpha-diversity indices suggested that plant species identity and flooding conditions were associated with rhizosphere microbial alpha diversity, which is consistent with frameworks emphasising host-associated filtering in microbiome assembly [63,64]. Shannon and Gini–Simpson diversity were lower in the flooded habitat for both hosts, whereas richness responses differed between *S. glauca* and *T. chinensis*, which may reflect the effects of hypoxia, pH fluctuations and nutrient availability on microbial community viability and competitive dynamics [65]. However, the responses of *T. chinensis* and *S. glauca* to environmental variation suggested different rhizosphere microbial response patterns that may reflect distinct life-history traits and adaptive strategies [66]. These results point to different habitat-associated response patterns between the two hosts. *T. chinensis* retained higher Shannon and Gini–Simpson diversity than *S. glauca* in the flooded habitat, whereas *S. glauca* showed larger changes in several predicted functional pathways between its two sampled habitats.

To further analyse habitat-associated differences in bacterial community structure, we ordinated all samples using NMDS based on Bray–Curtis dissimilarity. The results showed separation between the bacterial communities of *S. glauca* and *T. chinensis* under both flooded and non-flooded conditions. These findings are consistent with the interpretation that flooding is an important environmental factor associated with changes in bacterial community structure [67,68,69]. Moreover, plant species and flooding conditions can be associated with differences in bacterial community composition [70]. At the phylum level, Proteobacteria and Firmicutes were dominant bacterial groups in coastal saline–alkaline ecosystems, consistent with previous studies [71,72]. At the genus level, *Paucibacter* was a common dominant genus in both flooded and non-flooded soils, but its relative abundance varied across groups. Under flooded conditions, the abundance of Paucibacter in the rhizosphere soils of *T. chinensis* and *S. glauca* increased, possibly reflecting tolerance to oxygen-limited conditions or compatibility with flooded rhizosphere environments [73]. *Halomonas*, a typical halophilic genus, showed higher relative abundance mainly in non-flooded environments in this study, contrasting with previous findings that reported its enrichment in saline environments [74]. Overall, Shannon and Gini–Simpson diversity associated with *T. chinensis* was higher than that associated with *S. glauca*, whereas richness differences depended on the hydrological habitat and the metric examined, indicating differences in rhizosphere microbial assembly between these plant species [75]. Furthermore, part of the rhizosphere microbial community could not be accurately classified at known phylum or genus levels, reflecting the limitations of current microbial databases and leaving some taxa unresolved in saline–alkaline ecosystems [76,77]. The ecological roles of these unclassified sequences remain uncertain in coastal saline–alkaline ecosystems [78].

Comparative analyses using cluster heatmaps and LEfSe revealed differences in the rhizosphere bacterial taxa associated with *T. chinensis* and *S. glauca* under flooded habitats [79]. The rhizosphere microbial community of *S. glauca* showed closer clustering between flooded and non-flooded habitats, whereas that of *T. chinensis* exhibited greater divergence, indicating stronger habitat-associated differentiation in the bacterial community of *T. chinensis*. *Altererythrobacter* and *Novosphingobium*, both within the Sphingomonadaceae family, were widely distributed in *T. chinensis* rhizospheres but nearly absent in *S. glauca*. *Altererythrobacter* is typically associated with oligotrophic environments, and some members can degrade polycyclic aromatic hydrocarbons and lignin-derived compounds, potentially contributing to carbon turnover in the rhizosphere [80,81,82,83]. *Novosphingobium* exhibits metabolic versatility and potential in pollution remediation, with some members reported to participate in nitrogen fixation or aromatic-pollutant degradation [10,71,84,85,86,87].

Under flooded conditions, *S. glauca* was associated with enrichment of *Salinimicrobium*, *Sinomicrobium*, and *Pelagibius*, which have previously been reported in marine environments, although their functional roles in this system remain unclear [88,89,90]. In contrast, *T. chinensis* rhizospheres included genera containing potential pathogens (*Coxiella*, *Malaciobacter*) and autotrophic sulfur bacteria (*Desulfurivibrio*, *Sulfurivermis*) [91,92,93,94]. Non-flooded *T. chinensis* rhizospheres also contained *Desulfuromonas*, members of which can perform dissimilatory sulfur reduction, and *Legionella*, a genus containing potential pathogens [95]. These results suggest that host-associated filtering may contribute to differences in specific microbial taxa and community function under environmental stress through niche differentiation [96]. Phylogenetic analysis suggested that microbial communities associated with *T. chinensis* occupied distinct habitat-related niches under both flooded and non-flooded conditions, highlighting environmental differentiation and possible host-associated microbial filtering.

The network analysis provided additional exploratory evidence that the two host species were associated with different rhizosphere bacterial association structures. In the balanced display layer, *T. chinensis* showed more displayed nodes under non-flooded conditions, whereas the flooded *T. chinensis* network had the largest number of FDR-supported associations in the strict layer [97,98,99,100,101,102]. By contrast, *S. glauca* showed an increase in displayed node number under flooding but a decrease in density, average degree and FDR-supported associations. These patterns suggest differences in statistical association structure among plant–habitat groups [103,104,105,106,107]. The observed topological differences between host species and habitats are hypothesis-generating and require validation through expanded sampling, independent datasets or experimental manipulation.

Functional prediction using PICRUSt2 suggested species-specific differences in predicted functional potential [108]. In *S. glauca*, flooded and non-flooded habitats differed in the predicted abundance of carbon metabolism, ribosome-related and secondary-metabolite pathways, suggesting stress-associated shifts in predicted functional profiles that may be linked to nutrient mobilisation and protective metabolite production [30,109,110,111,112,113,114]. In contrast, *T. chinensis* showed relatively stable predicted functional profiles between hydrological habitats, suggesting a stability-oriented rhizosphere functional pattern.

These results indicate habitat-associated microbiome patterns that may contribute to halophyte adaptation to saline–alkaline environments and may inform future saline–alkali land restoration through microbial inoculation or plant–microbe synergy. This work provides an integrated analysis of rhizosphere bacterial community patterns under combined salinity–flooding conditions, offering insights relevant to coastal wetland management, ecological restoration and sustainable land-use strategies [115,116]. However, as functional predictions using PICRUSt2 are inferred from marker-gene data and reference genomes, they require experimental validation through metatranscriptomics or metabolomics. In addition, the present study was limited to two halophytes in a single tidal flat region. Future work should therefore expand to multi-species, multi-site, and temporal studies to further explore rhizosphere microbial adaptive strategies under saline–alkaline and flooded conditions.

## 4. Materials and Methods

### 4.1. Field Sampling and Site Characterisation

Two contrasting saline–alkaline field habitats were sampled: (1) a tidal-flat habitat subject to periodic flooding (37°43′54″ N, 118°58′3″ E; elevation: 2.48 m below sea level) and (2) a non-flooded saline–alkaline area (37°23′28″ N, 118°55′10″ E; elevation: 0.8 m above sea level), both described as sandy loam coastal tidal-flat soils [117]. The region has a warm temperate continental monsoon climate, with annual precipitation of 530–630 mm and a mean annual temperature of 11.5–12.4 °C [118]. Soil salinity in the Yellow River Delta coastal zone generally decreases with increasing distance from the sea [119].

Two dominant halophytes, *S. glauca* and *T. chinensis*, were selected as target species [120]. Four plant–habitat groups were defined: MC, *T. chinensis* in the flooded habitat; MH, *S. glauca* in the flooded habitat; NC, *T. chinensis* in the non-flooded habitat; and NH, *S. glauca* in the non-flooded habitat. For each plant–habitat group, five 1 m × 1 m quadrats were established, giving a total of 20 quadrats across the four plant–habitat groups.

Rhizosphere soil was collected following standardised procedures. Within a 10 cm radius around the base of each target plant, root–soil mixtures were collected to a depth of approximately 20 cm using a sterile shovel. After removal of large stones and plant debris, the root-associated soil was transferred into labelled sterile bags. Plant roots were gently separated, loosely attached soil was shaken off, and the tightly adhered rhizosphere soil layer, approximately 1 mm thick, was carefully collected using sterile instruments and transferred into labelled 1.5 mL sterile centrifuge tubes. All samples were transported to the laboratory on dry ice and stored at −80 °C until DNA extraction.

### 4.2. Soil Physicochemical Property Analysis

Soil physicochemical properties were determined using standardised soil analytical procedures. Soil organic carbon (SOC), total nitrogen (TN), electrical conductivity (EC), and pH were measured following the procedures described in Soil Agricultural Chemical Analysis. Total phosphorus (TP) was quantified according to the standard method LY/T 1232–2015. Stoichiometric ratios, including C:N, C:P, and N:P, were calculated from measured SOC, TN, and TP values.

### 4.3. Soil Microbial Genomic DNA Extraction

Genomic DNA was extracted from rhizosphere soil samples using the TGuide S96 Magnetic Bead Soil/Fecal Genomic DNA Extraction Kit (DP812; Tiangen Biotech, Beijing, China), following the manufacturer’s protocol. Briefly, approximately 0.25–0.50 g of rhizosphere soil was transferred into a 2 mL centrifuge tube, followed by the addition of 500 µL Buffer SA, 100 µL Buffer SC and 0.25 g grinding beads. Samples were homogenised by vortexing for 15 min or using a tissue-grinding homogeniser. After centrifugation at 12,000 rpm (approximately 13,400× *g*) for 1 min, the supernatant was transferred to a new 2 mL centrifuge tube for subsequent purification. To remove impurities and improve DNA purity, 200 µL Buffer SH was added to the supernatant, mixed thoroughly and incubated at 4 °C for 10 min. After centrifugation at 12,000 rpm for 3 min, the clarified supernatant was transferred to a new tube and mixed with 500 µL Buffer GFA. Magnetic bead suspension G was then added, and DNA was captured on magnetic beads. The beads were washed sequentially with protein-removal solution RD and wash buffer PWD. After air-drying at room temperature for 5–10 min to remove residual ethanol, DNA was eluted with 50–100 µL elution buffer TB at 56 °C for 5 min.

DNA concentration was measured using a dsDNA high-sensitivity fluorescence assay. DNA integrity and amplicon quality were evaluated by 1.8% agarose gel electrophoresis or LabChip GX Touch fragment analysis. Samples with correctly sized target bands and sufficient DNA quantity were retained for downstream PCR amplification and library construction.

### 4.4. DNA Amplification and High-Throughput Sequencing

The near full-length bacterial 16S rRNA gene was amplified using the primer pair 27F (5′-AGRGT TTGAT YNTGG CTCAG-3′) and 1492R (5′-TASGG HTACC TTGTT ASGAC TT-3′). PCR amplification was performed in a 20 µL reaction containing 2 µL genomic DNA, 6.5 µL nuclease-free water, 10 µL KOD ONE PCR Master Mix and 1.5 µL barcoded primer pair. The amplification program was as follows: initial denaturation at 95 °C for 2 min; 22 cycles of denaturation at 98 °C for 10 s, annealing at 55 °C for 30 s and extension at 72 °C for 1 min 30 s; and final extension at 72 °C for 2 min [121].

PCR products were checked using agarose gel electrophoresis or LabChip GX Touch fragment analysis. Qualified amplicons with the expected fragment size were purified and pooled according to product concentration and band quality. The pooled library was purified using magnetic beads, and sequencing libraries were prepared using the SMRTbell Prep Kit 3.0. Libraries were subjected to damage repair, end repair, adapter ligation, and AMPure PB bead purification. The final library was quantified using Qubit. Before sequencing, the library was bound with primer and polymerase using the Revio Polymerase Kit and loaded onto the PacBio sequencing system for single-molecule real-time sequencing.

### 4.5. Taxonomic Assignment and Diversity Analysis

Raw PacBio reads were processed to generate high-quality circular consensus sequences (CCS) [122]. Barcode demultiplexing was performed according to sample-specific barcodes, and primer sequences were removed before downstream analysis. Cutadapt was used for primer trimming and quality filtering [123]. Chimeric sequences were identified and removed using UCHIME [124]. High-quality non-chimeric sequences were clustered into operational taxonomic units (OTUs) at 97% sequence similarity using USEARCH, and low-abundance OTUs were removed before downstream analyses [125,126]. Representative OTU sequences were taxonomically assigned against the SILVA database using a naïve Bayes classifier implemented in QIIME 2, with a confidence threshold of 0.70 [127].

Alpha-diversity metrics were calculated from the OTU abundance table, including observed species richness, ACE, Chao1, Shannon diversity, Gini–Simpson diversity and Good’s coverage [128]. Four plant–habitat groups were defined as follows: NH, non-flooded *S. glauca*; MH, flooded *S. glauca*; NC, non-flooded *T. chinensis*; and MC, flooded *T. chinensis*. For each alpha-diversity metric, a two-factor sample-level model was fitted with plant species, hydrological habitat and their interaction as fixed factors. Beta diversity was evaluated using Bray–Curtis dissimilarity, binary Jaccard distance, Kulczynski distance, and Euclidean distance after Hellinger transformation to assess microbial community dissimilarity among samples [128]. Taxonomic differences between groups were identified with linear discriminant analysis effect size (LEfSe), applying an LDA score threshold of 4.0. PERMANOVA was used to evaluate the contributions of plant species and hydrological habitat to differences in community composition. Candidate biomarker taxa were additionally ranked using random forest feature importance.

### 4.6. Predicted Functional Profiling

Predicted bacterial functional profiles were inferred from the 16S rRNA gene OTU table using PICRUSt2 [108]. Predicted KEGG pathway profiles were compared between flooded and non-flooded habitats separately for *S. glauca* and *T. chinensis*.

### 4.7. Microbial Association Network Construction

Treatment-specific microbial consensus association networks were constructed from the 16S rRNA gene abundance table after matching sample identifiers with the sample metadata. The analysis was performed at the genus level. For each treatment group (NH, MH, NC and MC), taxa were filtered according to group-wise total abundance ≥ 40 and prevalence ≥ 20% across the five biological replicates, and the top 240 genera by total abundance were retained for network inference. To reduce compositional scaling effects, genus-level count profiles were transformed using a centred log-ratio (CLR) transformation with a pseudocount of 0.5. Pairwise associations among retained taxa were first estimated using Pearson correlation coefficients calculated from CLR-transformed abundance profiles. A secondary Spearman rank correlation analysis on the CLR-transformed data was used as a sign-consistency screen. Only taxon pairs with consistent association signs between the primary and secondary analyses were retained for the consensus workflow. Leave-one-out sign consistency was calculated as a stability diagnostic, and only edges with sign consistency ≥ 0.60 were retained. Multiple-testing correction was performed using the Benjamini–Hochberg false discovery rate (FDR) procedure, with FDR < 0.05 defining the strict statistical layer. The balanced consensus display layer retained edges with |r| ≥ 0.65 (Pearson) and concordant Spearman sign, irrespective of FDR status, to ensure visual completeness.

Undirected weighted networks were generated after removing self-loops. Nodes represented retained genera; unclassified taxa were preserved as genus-level identifiers when family-level assignment was unavailable. Edges represented statistical associations inferred from the consensus workflow and were drawn with three evidence classes: solid lines for FDR-supported and consistency-filtered edges, dashed lines for consistency-supported but non-FDR edges, and dotted lines for context-only taxon pairs below the display threshold. Node attributes included phylum-level taxonomy, treatment-specific abundance, degree and module membership. Context-only taxa with degree zero were retained as faint halo nodes in the display figures to provide community background, but these nodes were not interpreted as having inferred associations. Network modules were identified using weighted Louvain community optimisation. Network layouts were generated using the Fruchterman–Reingold algorithm with context halo rendering. Network visualisation and summary statistics were performed in R (v4.3+) using igraph, tidygraph, ggraph, ggplot2, cowplot, patchwork, showtext and related dependencies. Sensitivity-ready inputs for SPIEC-EASI, SparCC and NetCoMi were exported but not formally fitted within this study.

### 4.8. Phylogenetic Analysis

Phylogenetic analysis was performed to describe evolutionary relationships and community structure patterns among rhizosphere bacterial communities. The 500 most abundant OTUs were aligned with MAFFT v7.490 using default parameters. An approximately maximum-likelihood phylogenetic tree was generated using FastTree v2.1.11. Phylogenetic trees were visualised in R v4.2.3 using the ggtree v3.15.0, employing circular, rectangular, and semicircular layouts to optimise data presentation [129]. Multiple data layers, including taxonomic classifications, abundance matrices, and environmental metadata were integrated to enhance interpretation. Major bacterial phyla were colour-coded on branches, while tip points were distinguished by sampling site with distinct shapes, and sizes proportional to relative abundance. Environmental annotations were displayed as ring-based heatmaps showing site-specific abundance patterns, complemented by outer bar plots representing relative abundance distributions.

### 4.9. Data Visualisation

Alpha-diversity metrics were visualised with box plots containing statistical annotations, and rarefaction curves were generated to assess sequencing depth sufficiency. Taxonomic composition was displayed using stacked bar plots and hierarchical clustering heatmaps. Predicted functional pathway comparisons were illustrated with forest plots to show differences between conditions. The results of the association network analysis were presented in both phylum-coloured and module-coloured formats to show microbial association patterns. Biomarker identification was performed with LEfSe, and results were visualised as cladograms and LDA score plots.

## 5. Conclusions

This study provides exploratory evidence that two dominant halophytes in the Yellow River Delta are associated with distinct rhizosphere bacterial community patterns under saline–alkaline and hydrological habitat contrasts. Hydrological habitat conditions and plant identity together contributed to differences in rhizosphere microbial community composition. *T. chinensis* generally harboured higher bacterial diversity, whereas *S. glauca* and *T. chinensis* showed contrasting patterns in predicted functional potential and exploratory microbial association networks. PICRUSt2-based analysis suggested that predicted functional profiles were more variable between hydrological habitats in the *S. glauca* rhizosphere, while no predicted pathway shifts were significant after multiple-testing correction in the *T. chinensis* rhizosphere. These contrasting habitat-associated patterns provide a basis for future studies of plant–microbiome relationships along saline–alkaline and hydrological gradients and may inform wetland restoration research.

## Data Availability

The raw sequence data generated in this study have been deposited in the NCBI Sequence Read Archive (SRA) under BioProject accession number PRJNA1276525. The dataset comprises 20 full-length 16S rRNA gene amplicon samples sequenced on the PacBio Sequel II platform, with individual BioSample accessions ranging from SAMN51492768 to SAMN51492787 and corresponding SRA experiment accessions SRX30562599 to SRX30562618. The complete dataset, including all raw sequencing reads and associated metadata, is accessible through the SRA Study accession SRP621201. The data are available through NCBI BioProject PRJNA1276525 at https://www.ncbi.nlm.nih.gov/bioproject/PRJNA1276525 (accessed on 25 May 2026).

## Supplementary Materials

The following supporting information can be downloaded at: https://www.mdpi.com/article/10.3390/plants15172565/s1, Table S1. Descriptive statistics of soil physicochemical properties under flooded and non-flooded saline–alkaline rhizosphere habitats.
